# Supplementary material for: Antifungal Activity of Guiera senegalensis: From the Chemical Composition to the Mitochondrial Toxic Effects and Tyrosinase Inhibition
Source: Antibiotics (Basel). 2023 May 8;12(5):869. doi: 10.3390/antibiotics12050869 (PMC10215606; doi:10.3390/antibiotics12050869)
Supplement: Supplementary file 1 [file antibiotics-12-00869-s001.zip › antibiotics-2380068-supplementary.pdf]

**Supplementary material**

**Table S1.** Linear regression equation analysis, limit of detection (LOD) and limit of quantification (LOQ) for external standards.

| Standard [1] | regression equation | | | linearity range (μg/mL) | LOD (μg/mL) | LOQ (μg/mL) |
|---|---|---|---|---|---|---|
| | slope ($\sigma$) | intercept ($b$) | $R^2$ (n = 3) | | | |
| gallic acid | 63.644 | −156.840 | 0.9998 | 6.25–200 | 0.116 | 0.352 |
| myricetin-3-*O*-glucoside | 16.314 | −79.310 | 0.9997 | 12.5–400 | 2.515 | 7.621 |
| myricitrin | 38.099 | −376.870 | 0.9999 | 25–800 | 0.973 | 2.949 |
| quercetin-3-*O*-galactoside | 57.521 | −431.470 | 0.9953 | 6.25–200 | 0.122 | 0.371 |
| quercetin-3-*O*-glucoside | 25.381 | −46.102 | 0.9996 | 4.69–150 | 0.469 | 1.422 |
| quercetin-3-*O*-arabinoside | 20.192 | 20.251 | 0.9998 | 2.5–80 | 0.158 | 0.479 |
| quercetin-3-*O*-rhamnoside | 28.511 | −38.083 | 0.9997 | 2.81–90 | 0.398 | 1.205 |
| myricetin | 43.683 | −149.500 | 0.9996 | 6.25–200 | 0.311 | 0.943 |
| kaempferol-3-*O*-glucoside | 29.610 | −26.566 | 0.9999 | 6.25–200 | 1.099 | 3.330 |
| quercetin | 39.213 | −18.603 | 0.9985 | 0.78–25 | 0.085 | 0.257 |
| hesperetin | 63.191 | 0.981 | 0.9991 | 1.56–50 | 0.089 | 0.271 |
| isorhamnetin | 14.284 | 0.782 | 0.9992 | 0.94–30 | 0.078 | 0.237 |
| mangiferin | 19.527 | 1394.000 | 0.9961 | 500–2000 | 370.744 | 1123.466 |
| chlorophyll *a* | 7.292E-05 | 8.000E+06 | 0.9991 | 6.25–1600 | 2.181 | 6.609 |
| chlorophyll *b* | 9.610E-05 | 2.000E+06 | 0.9976 | 6.25–200 | 1.426 | 4.321 |

[1] Myricetin-3-*O*-glucoside (**14**), myricetin-3-*O*-rhamnoside (**19**), quercetin-3-*O*-galactoside (**20**), quercetin-3-*O*-glucoside (**21**), quercetin-3-*O*-arabinoside (**23**), quercetin-3-*O*-rhamnoside (**24**), myricetin (**25**), quercetin (**28**), hesperetin (**32**) and isorhamnetin (**33**) were quantified as themselves. Gallic acid was used to quantify compounds **1**-**10**; myricetin-3-*O*-glucoside for compounds **11**-**13**, **15** and **18**; quercetin-3-*O*-galactoside for compounds **16** and **17**; quercetin-3-*O*-arabinoside for compound **22**; kaempferol-3-*O*-glucoside for compounds **26**, **27**, **29**-**31;** and mangiferin for compound **34** (Figure 1 and Table 2). Chlorophylls *a* (**4**) and *b* (**2**) were quantified as themselves. Chlorophyll *a* was used to quantify compounds **1**, **3-5**, **7** and **10;** chlorophyll *b* for compounds **2**, **6**, **8-9** and **11** (Figure 3 and Table 3).

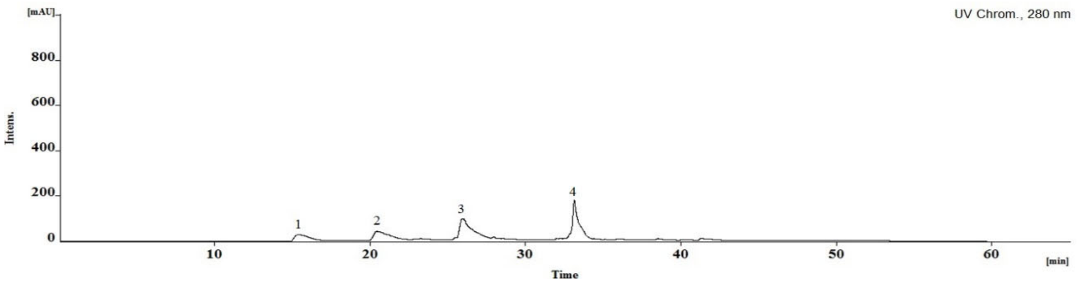


**Figure S1.** HPLC-UV profile of the alkaloid-targeted *G. senegalensis* leaves extract, using a reverse phase Luna C18 (Phenomenex, California, USA) with spectra acquisition in continuous scan mode from 200 to 600 nm, and the chromatogram recorded at 280 nm. The solvent system was composed by $H_2O$ (pH 2 with $H_3PO_4$) (elunt A) and acetonitrile:water (90:10, v/v) (eluent B) and the gradient used was: 0 min – 90% B, 15 min – 50% B, 17 – 35 min – 90% B.
